# Apoptosis Induction and Gene Expression Profile Alterations of Cutaneous T-Cell Lymphoma Cells following Their Exposure to Bortezomib and Methotrexate

**DOI:** 10.1371/journal.pone.0170186

**Published:** 2017-01-20

**Authors:** Vassiliki Mpakou, Evangelia Papadavid, Frieda Kontsioti, Eugene Konsta, Miriam Vikentiou, Aris Spathis, Sotiris Papageorgiou, Diamantina Vasilatou, Konstantinos Gkontopoulos, Efthimia Mpazani, Petros Karakitsos, Dimitrios Rigopoulos, George Dimitriadis, Vasiliki Pappa

**Affiliations:** 1 Second Dept. of Internal Medicine and Research Institute, Attikon University General Hospital, Rimini 1, Haidari, Athens, Greece; 2 Second Department of Dermatology, Attikon University General Hospital, Rimini 1, Haidari, Athens, Greece; 3 First Department of Dermatology, A. Syggros University Hospital, Athens University, Medical School, Greece; 4 Department of Cytopathology, Attikon University General Hospital, Athens, Greece; University of Hawaii System, UNITED STATES

## Abstract

Mycosis fungoides (MF) and its leukemic variant Sézary syndrome (SS) comprise the majority of CTCL, a heterogenous group of non-Hodgkins lymphomas involving the skin. The CTCL’s resistance to chemotherapy and the lack of full understanding of their pathogenesis request further investigation. With the view of a more targeted therapy, we evaluated *in vitro* the effectiveness of bortezomib and methotrexate, as well as their combination in CTCL cell lines, regarding apoptosis induction. Our data are of clinical value and indicate that the bortezomib/methotrexate combinational therapy has an inferior impact on the apoptosis of CTCL compared to monotherapy, with bortezomib presenting as the most efficient treatment option for SS and methotrexate for MF. Using PCR arrays technology, we also investigated the alterations in the expression profile of genes related to DNA repair pathways in CTCL cell lines after treatment with bortezomib or methotrexate. We found that both agents, but mostly bortezomib, significantly deregulate a large number of genes in SS and MF cell lines, suggesting another pathway through which these agents could induce apoptosis in CTCL. Finally, we show that SS and MF respond differently to treatment, verifying their distinct nature and further emphasizing the need for discrete treatment approaches.

## Introduction

Cutaneous T-cell lymphomas (CTCLs) are rare skin malignancies, comprising a heterogeneous group of non-Hodgkin lymphomas derived from skin-homing mature T-cells [1]. Mycosis fungoides (MF) and Sézary syndrome (SS) are considered as the commonest CTCL types and together account for approximately 50% of all CTCL cases [2, 3]. MF is considered as the commonest type of CTCL and is initially characterized by patches and infiltrated plaques on the skin which eventually evolve into tumors [4, 5]. SS is the leukemic variant of MF and is characterized by erythroderma, lymphadenopathy and the presence of a malignant T-cell clone in the peripheral blood and the skin [2, 5].

Advances in therapy for CTCL are mostly focused on the development of novel pharmacological targets but in most cases the response is short and the survival rate is not significantly improved [2, 4, 6, 7]. Combinational therapy of known pharmaceutical agents is another treatment option for CTCL patients, which could be beneficial and lead to better responses compared to monotherapy [8].

Bortezomib, a dipeptide boronic acid analog, is a proteasome inhibitor with antitumor activity [9, 10], which reversibly inhibits the chymotryptic activity of the 20S subunit of the proteasome and leads to several downstream effects, including activation of p53, inhibition of NF-kB and accumulation of pro-apoptotic proteins [11]. Bortezomib was approved in 2003 by the FDA for the treatment of multiple myeloma and for relapsed or refractory mantle cell lymphoma [12, 13] and a vast number of reports and clinical trials reveal that it can also be used for the treatment of solid tumors, alone or in combination [14–16]. Bortezomib has shown promising results in patients with relapsed or refractory CTCL [17–20]. Nevertheless, its exact molecular mechanism of action in CTCL is not fully understood [21].

Methotrexate is an antimetabolite, a structural analogue of folic acid, which acts as an inhibitor of the enzyme dihydrofolate reductase (DHFR), leading to the depletion of tetrahydrofolate cofactors that are required for DNA and RNA synthesis [22], and therefore to the induction of cell death by secondary genotoxic effects or apoptosis [23]. Methotrexate is an essential anticancer agent particularly for human leukemia, severe psoriasis and the treatment of some solid tumors [24, 25] and is already used for the treatment of CTCL patients, alone or in combination with other agents [26–31].

We investigated the ability of bortezomib and methotrexate to induce apoptotic cell death in CTCL cell lines, alone or in combination, in order to evaluate each agent’s effectiveness in overcoming the denoted CTCLs’ apoptotic resistance and determine whether or not combinational therapy presents a higher apoptotic efficiency compared to monotherapy. We further investigated the alterations in the expression profile of selected genes involved in the DNA repair signalling in CTCL cell lines after treatment with bortezomib or methotrexate, aiming at a better understanding of their pathogenesis and the mechanisms of action of the aforementioned pharmaceutical agents in CTCL.

## Materials and Methods

### Cell lines and culture

Human CTCL cell lines Hut-78, SeAx and Myla were a generous gift from Dr Margarita Sánchez-Beato (Health Research Institute, Hospital Universitario Puerta de Hierro Majadahonda, Madrid, Spain). Cells were cultured in RPMI 1640, supplemented with 10% fetal bovine serum and 1% penicillin/streptomycin. All cell lines were treated with bortezomib (10nmol/L), methotrexate (10μM) and their combination (bortezomib 10nmol/L and methotrexate 10μM), at 37°C in a humidified atmosphere with 5% CO_2_, for 24 hours.

### Flow cytometric analysis of cell apoptosis

Hut-78 (SS), SeAx (SS) and Myla (MF) cells were cultured with or without addition of the drugs at the indicated concentrations for 24h. Apoptosis was analyzed using the Annexin V/PI assay, as previously described [32].

### Human DNA repair signalling pathway detection by RT^2^ Profiler PCR Arrays

Total RNA extraction and purification from untreated and drug-treated CTCL cell lines was performed using the RNeasy MinElute kit (Qiagen Ltd., Hilden, Germany), according to the manufacturer’s instructions. DNase enzyme digestion was performed to exclude genomic DNA contamination. RNA was quantified using NanoDrop 1000 spectrophotometer (Thermo Scientific, Waltham, USA). Total RNA with 260/280 ratio of 2.0 or more was utilized for real-time PCR quantification by RT^2^ Profiler^TM^ PCR arrays (SABiosciences Corp.)

Real-time PCR quantification was performed on a Corbett Rotor Gene 6000 system (Qiagen Ltd.) using an RT^2^ Profiler custom PCR Array consisting of a panel of 41 genes involved in DNA Damage Signaling and DNA Repair mechanisms, including two “housekeeping genes”. cDNAs were synthesized from 0.8 μgr of total RNA using the commercial RT^2^ First Strand Kit (Qiagen, Ltd.), according to the manufacturer’s instructions. The obtained cDNA was mixed with RT^2^ qPCR master-mix, containing SYBR green (SA Biosciences). The mixture was subsequently added into each well of the RT^2^ Profiler custom PCR Array and qPCR was performed according to the manufacturer’s instructions. All runs were performed in duplicate. Data were analyzed using the integrated web-based automated software for RT^2^ Profiler PCR Array Data analysis (RT^2^ Profiler PCR Array Data analysis version 3.5, GeneGlobe Data Analysis) available through SABiosciencies. Fold changes in each gene expression were calculated using the ^ΔΔ^C_t_ method and housekeeping gene controls were used for normalization of the results. A two-fold or greater change in expression was considered significant. Fold-change values less than one were indicative of down-regulation of the gene expression and fold-change values greater than one were indicative of an up-regulation.

### Statistical analysis

Statistical analyses were performed with SPSS software (version 16.0; SPSS, Inc., Chicago, IL., USA). All experiments were performed three times in each individual sample and the results were given as the mean value of the three. One-way Anova and LSD/ Bonferroni methods were used to compare the means among more than two different groups. Descriptive statistics, such as mean values and SD, were calculated. Data were reported as mean ± standard deviation (SD) of the mean. A two-sided p value <0.05 was considered statistically significant.

## Results

### Bortezomib, methotrexate, and their combination induce apoptosis in SS cell lines

**Fig 1:**
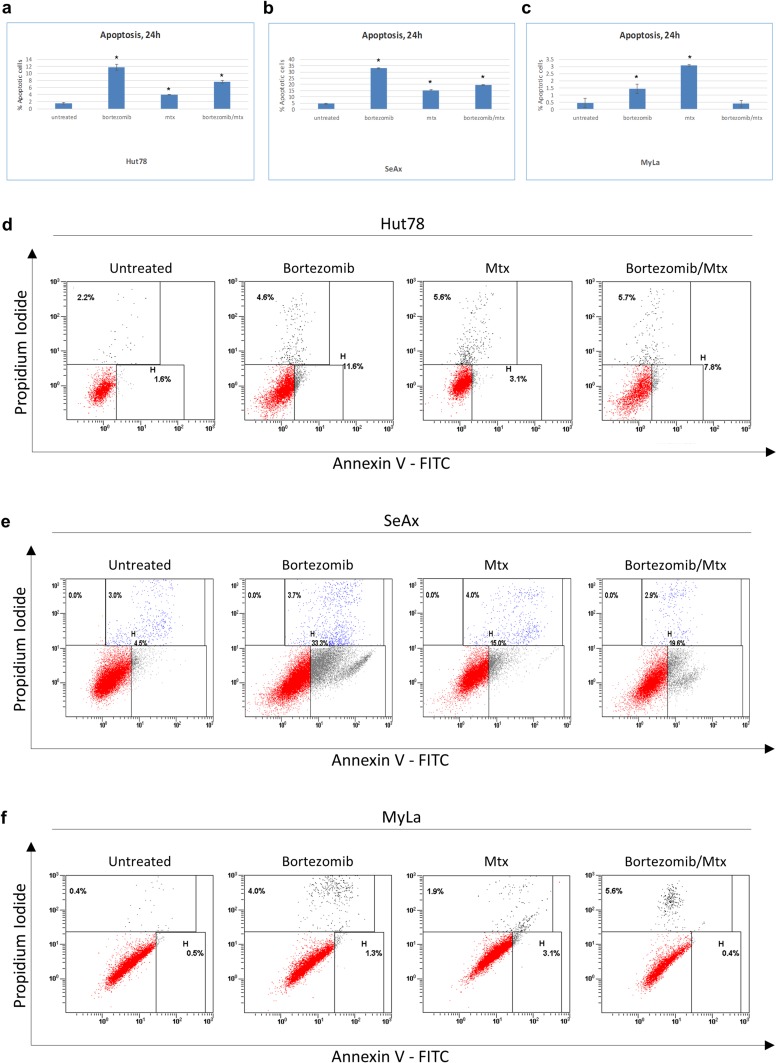
Apoptosis of CTCL cell lines after exposure to bortezomib and/or methotrexate. Percentage of Annexin-V positive untreated cells and cells treated with 10nmol/L bortezomib, 10μM methotrexate and their combination for 24h for (a) Hut-78 (b) SeAx and (c) MyLa cell lines. Graphs show the mean ± S.D. of three independent experiments. Representative dot plots of the Annexin-V and PI assay on Hut-78 (d), SeAx (e) and MyLa (f) cell lines are shown. Statistically significant differences (p<0.05) were determined by Bonferroni analysis.

We initially analyzed the apoptotic effects of bortezomib and/or methotrexate on the SS-derived Hut-78 and SeAx cells (Fig 1). We found that after 24h of drug exposure, Hut-78 cells responded with enhanced apoptosis when treated with either agent or their combination, compared to untreated cells. The higher apoptotic effect was observed after treatment with bortezomib, followed by the two drugs’ combination (11.76% and 7.61% vs 1.53% respectively, p = 0.000) and methotrexate (3.98% vs 1.53%, p = 0.000) (Fig 1A and 1D), (data shown as mean ± SD derived from three independent experiments). SeAx cells presented a similar apoptotic profile to Hut-78 cells, exhibiting enhanced apoptotic rates after all treatments, with bortezomib showing the higher apoptotic rate, followed by the two agents’ combination and methotrexate (33.0%, 19.48% and 15.31% vs 4.45, respectively p = 0.000) (Fig 1B and 1E).

### Bortezomib and methotrexate but not their combination induce apoptosis in the MF cell line MyLa

The MF-derived cell line MyLa was treated with bortezomib, methotrexate or their combination for 24h. FACS analysis after Annexin V/PI assay (Fig 1C and 1F) revealed that both agents enhanced the apoptosis of MyLa cells compared to control, unlike combinational treatment where no statistically significant change was observed. Between the two drugs, methotrexate had the higher apoptotic impact on MyLa cells (3.09% and 1.4% vs 0.44% respectively, p = 0.000 and p<0.01).

### Bortezomib-treatment significantly affects DNA repair signaling in SS cell lines

**Fig 2:**
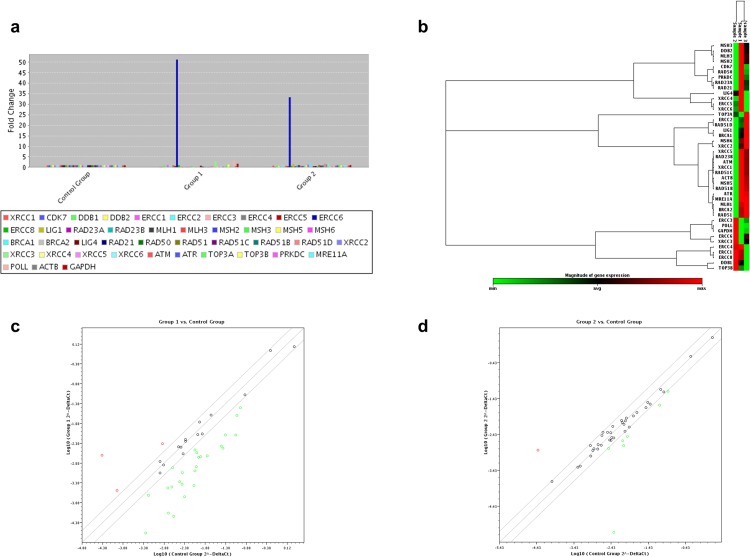
Differential gene expression in Hut-78 cell line determined by RT^2^ Profiler PCR arrays analysis after treatment with bortezomib and methotrexate. (a) Bar chart of the DNA repair related genes distribution after treatment with bortezomib (Group 1) and methotrexate (Group 2), (b) heat map of gene distribution after non-supervised hierarchical clustering of DNA repair related genes showing co-regulated genes across untreated (Sample 1), bortezomib-treated (Sample 2) and methotrexate-treated Hut-78 cells (Sample 3), (c) scatter plot of gene distribution after bortezomib-treatment (Group 1) and (d) after methotrexate-treatment (Group 2). In (a) each bar represents one gene. In (b) each box represents one gene, with minimum expression indicated as green, maximum as red and average as black. In the scatter plots (c) and (d) each circle represents one gene and up-regulated, down-regulated and unchanged genes are indicated as red, green and black, respectively.

**Fig 3:**
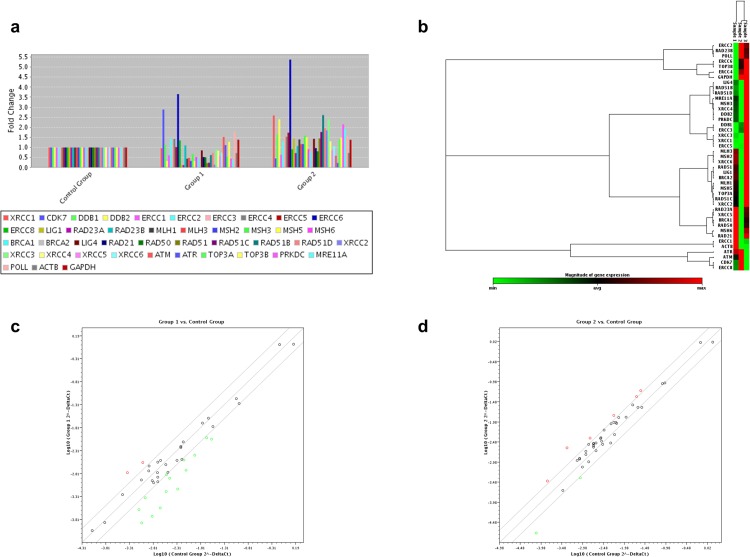
Differential gene expression in SeAx cell line determined by RT^2^ Profiler PCR arrays analysis after treatment with bortezomib and methotrexate. (a) Bar chart of the DNA repair related genes distribution after treatment with bortezomib (Group 1) and methotrexate (Group 2), (b) heat map of gene distribution after non-supervised hierarchical clustering of DNA repair related genes showing co-regulated genes across untreated (Sample 1), bortezomib-treated (Sample 2) and methotrexate-treated SeAx cells (Sample 3), (c) scatter plot of gene distribution after bortezomib-treatment (Group 1) and (d) after methotrexate-treatment (Group 2). In (a) each bar represents one gene. In (b) each box represents one gene, with minimum expression indicated as green, maximum as red and average as black. In the scatter plots (c) and (d) each circle represents one gene and up-regulated, down-regulated and unchanged genes are indicated as red, green and black, respectively.

To identify the potential effect of bortezomib on the cellular pathways related to DNA damage and repair of SS cell lines (Hut-78 and SeAx), differences in the mRNA levels of selected genes were examined using a custom RT^2^ Profiler PCR array by comparing bortezomib-treated cells with control (untreated) cells. We found that bortezomib had a great effect in the expression profile of genes involved in all DNA repair pathways examined, in both Hut-78 (Table 1) and SeAx (Table 2) cells, with Hut-78 being affected the most (Figs 2 and 3). Specifically, in Hut-78 cell line, three genes, *ERCC6*, *POLL* and *XRCC3* were up-regulated more than 2-fold after treatment, with *ERCC6* presenting the most significant change (higher than 50-fold) (Fig 2A–2C). *ERCC6* was also up-regulated more than 2-fold in SeAx cells, but in a rather moderate way compared to Hut-78, along with *CDK7* gene (Fig 3A–3C). In both cell lines, we observed a significant down-regulation of a large number of genes. A total of 25 genes were more than 2-fold down-regulated in Hut-78 compared to untreated cells (Fig 2A–2C), such as genes involved in the Double-Strand Break (DSB) repair mechanism (*BRCA1*, *BRCA2*, *XRCC2*, *PRKDC*, *RAD51*, *RAD51C*, *RAD50*, *MRE11A*, *XRCC5*, *RAD21*, *ATM*, *XRCC4*), the Mismatch Repair mechanism (MMR) (*MSH6*, *MSH2*, *MLH1*, *MSH5*, *MLH3*), the Nucleotide Excision Repair (NER) mechanism (*RAD23B*, *CDK7*, *RAD23A*, *DDB2*, *LIG1*) and the Single-Strand Break Repair (SSDR) mechanism (*XRCC1*). *ATR*, *XRCC2*, *BRCA2* and *BRCA1* genes presented the highest changes, with more than a 20-fold down-regulation in their expression, compared to control. Similarly, in bortezomib-treated SeAx cells, a total of 14 genes involved in all the DNA repair pathways tested were down-regulated more than 2-fold compared to control (DSB: *XRCC2*, *BRCA2*, *RAD51*, *RAD51C*, *MRE11A*, BRCA1, *PRKDC*; MMR: *MSH5*, *MSH2*, *MLH1*, *MLH3*; NER: *LIG1*, *RAD23A*, *DDB2*) with *BRCA2*, *XRCC2*, *LIG1* and *RAD23A* genes presenting the most significant alterations (down-regulation of more than 6-fold change) (Fig 3A–3C).

**Table 1:** Fold changes of transcription in bortezomib- and methotrexate-treated Hut-78 cell line compared to that of untreated Hut78 cell line.

Gene Symbol	Name of gene	Fold up- or down-regulation (Bortezomib-treated vs control)	Fold up- or down-regulation (Methotrexate-treated vs control)
XRCC1	X-ray repair complementing defective repair in Chinese hamster cells 1	-3,387	-1,1647
CDK7	Cyclin-dependent kinase 7	-7,5685	-4,4076
DDB1	Damage-specific DNA binding protein 1, 127kDa	1,1728	-1,1975
DDB2	Damage-specific DNA binding protein 2, 48kDa	-6,0629	-1,5911
ERCC1	Excision repair cross-complementing rodent repair deficiency, complementation group 1 (includes overlapping antisense sequence)	1,181	-630,3459
ERCC2	Excision repair cross-complementing rodent repair deficiency, complementation group 2	-1,0867	1,1647
ERCC3	Excision repair cross-complementing rodent repair deficiency, complementation group 3 (xeroderma pigmentosum group B complementing)	1,4743	1,0943
ERCC4	Excision repair cross-complementing rodent repair deficiency, complementation group 4	-1,014	-1,3195
ERCC5	Excision repair cross-complementing rodent repair deficiency, complementation group 5	-1,5692	-1,8921
ERCC6	Excision repair cross-complementing rodent repair deficiency, complementation group 6	51,2685	33,3589
ERCC8	Excision repair cross-complementing rodent repair deficiency, complementation group 8	1,0644	-1,5052
LIG1	Ligase I, DNA, ATP-dependent	-2,1287	1,6245
RAD23A	RAD23 homolog A (S. cerevisiae)	-7,0128	-2,0994
RAD23B	RAD23 homolog B (S. cerevisiae)	-11,3924	-1,2397
MLH1	MutL homolog 1, colon cancer, nonpolyposis type 2 (E. coli)	-8,8152	1,0718
MLH3	MutL homolog 3 (E. coli)	-4,9246	-1,454
MSH2	MutS homolog 2, colon cancer, nonpolyposis type 1 (E. coli)	-9,2535	-1,5801
MSH3	MutS homolog 3 (E. coli)	-1,6818	-1,2658
MSH5	MutS homolog 5 (E. coli)	-5,2416	-1,1096
MSH6	MutS homolog 6 (E. coli)	-19,4271	1,2924
BRCA1	Breast cancer 1, early onset	-36,7583	1,9053
BRCA2	Breast cancer 2, early onset	-23,1029	1,1567
LIG4	Ligase IV, DNA, ATP-dependent	-1,2658	-1,9319
RAD21	RAD21 homolog (S. pombe)	-2,9282	-1,7171
RAD50	RAD50 homolog (S. cerevisiae)	-5,2416	-3,1383
RAD51	RAD51 homolog (S. cerevisiae)	-9,3827	1,1329
RAD51C	RAD51 homolog C (S. cerevisiae)	-7,3615	-1,2142
RAD51B	RAD51 homolog B (S. cerevisiae)	-6,0629	-1,0943
RAD51D	RAD51 homolog D (S. cerevisiae)	-1,4044	1,7411
XRCC2	X-ray repair complementing defective repair in Chinese hamster cells 2	-22,1618	1,4948
XRCC3	X-ray repair complementing defective repair in Chinese hamster cells 3	2,8679	1,9053
XRCC4	X-ray repair complementing defective repair in Chinese hamster cells 4	-2,6759	-3,0314
XRCC5	X-ray repair complementing defective repair in Chinese hamster cells 5 (double-strand-break rejoining)	-3,7581	-1,1975
XRCC6	X-ray repair complementing defective repair in Chinese hamster cells 6	-1,7901	-2,1287
ATM	Ataxia telangiectasia mutated	-2,7132	-1,1567
ATR	Ataxia telangiectasia and Rad3 related	-20,8215	-1,0353
TOP3A	Topoisomerase (DNA) III alpha	1,1487	1,434
TOP3B	Topoisomerase (DNA) III beta	1,3755	-1,1251
PRKDC	Protein kinase, DNA-activated, catalytic polypeptide	-11,0809	-3,0525
MRE11A	MRE11 meiotic recombination 11 homolog A (S. cerevisiae)	-4,8568	-1,007
POLL	Polymerase (DNA directed), lambda	3,3636	1,0943
ACTB	Actin, beta	-1,7532	-1,0718
GAPDH	Glyceraldehyde-3-phosphate dehydrogenase	1,7532	1,0718

**Table 2:** Fold changes of transcription in bortezomib- and methotrexate-treated SeAx cell line compared to that of untreated SeAx cell line.

Gene Symbol	Name of gene	Fold up- or down-regulation (Bortezomib-treated vs control)	Fold up- or down-regulation (Methotrexate-treated vs control)
XRCC1	X-ray repair complementing defective repair in Chinese hamster cells 1	-1,0353	2,5937
CDK7	Cyclin-dependent kinase 7	2,8879	-2,1962
DDB1	Damage-specific DNA binding protein 1, 127kDa	1,1408	1,6415
DDB2	Damage-specific DNA binding protein 2, 48kDa	-3,0525	2,4033
ERCC1	Excision repair cross-complementing rodent repair deficiency, complementation group 1 (includes overlapping antisense sequence)	-1,6472	-1,5637
ERCC2	Excision repair cross-complementing rodent repair deficiency, complementation group 2	1,4142	1,3059
ERCC3	Excision repair cross-complementing rodent repair deficiency, complementation group 3 (xeroderma pigmentosum group B complementing)	1,1251	1,5105
ERCC4	Excision repair cross-complementing rodent repair deficiency, complementation group 4	1,4241	1,5316
ERCC5	Excision repair cross-complementing rodent repair deficiency, complementation group 5	1,014	1,7351
ERCC6	Excision repair cross-complementing rodent repair deficiency, complementation group 6	3,6553	5,3703
ERCC8	Excision repair cross-complementing rodent repair deficiency, complementation group 8	1,3472	-1,0981
LIG1	Ligase I, DNA, ATP-dependent	-7,9447	1,4389
RAD23A	RAD23 homolog A (S. cerevisiae)	-8,0556	-1,3899
RAD23B	RAD23 homolog B (S. cerevisiae)	1,1019	1,0681
MLH1	MutL homolog 1, colon cancer, nonpolyposis type 2 (E. coli)	-2,2658	1,3996
MLH3	MutL homolog 3 (E. coli)	-2,0562	1,1607
MSH2	MutS homolog 2, colon cancer, nonpolyposis type 1 (E. coli)	-3,0314	1,1688
MSH3	MutS homolog 3 (E. coli)	-1,4948	1,5856
MSH5	MutS homolog 5 (E. coli)	-3,0738	1,5
MSH6	MutS homolog 6 (E. coli)	-1,9185	-1,0981
BRCA1	Breast cancer 1, early onset	-2,2815	-1,3149
BRCA2	Breast cancer 2, early onset	-6,774	1,4093
LIG4	Ligase IV, DNA, ATP-dependent	-1,1728	1,429
RAD21	RAD21 homolog (S. pombe)	-1,9319	-1,0317
RAD50	RAD50 homolog (S. cerevisiae)	-1,9588	-1,2354
RAD51	RAD51 homolog (S. cerevisiae)	-4,5631	1,4489
RAD51C	RAD51 homolog C (S. cerevisiae)	-4,1699	1,7715
RAD51B	RAD51 homolog B (S. cerevisiae)	-1,6133	2,6117
RAD51D	RAD51 homolog D (S. cerevisiae)	-1,3755	1,9521
XRCC2	X-ray repair complementing defective repair in Chinese hamster cells 2	-7,21	1,8468
XRCC3	X-ray repair complementing defective repair in Chinese hamster cells 3	-1,1173	2,42
XRCC4	X-ray repair complementing defective repair in Chinese hamster cells 4	-1,1892	1,3059
XRCC5	X-ray repair complementing defective repair in Chinese hamster cells 5 (double-strand-break rejoining)	-1,7171	-1,1688
XRCC6	X-ray repair complementing defective repair in Chinese hamster cells 6	-1,3379	1,0681
ATM	Ataxia telangiectasia mutated	1,5157	-1,7112
ATR	Ataxia telangiectasia and Rad3 related	1,1173	-4,3924
TOP3A	Topoisomerase (DNA) III alpha	-1,8661	1,3996
TOP3B	Topoisomerase (DNA) III beta	1,2834	1,4794
PRKDC	Protein kinase, DNA-activated, catalytic polypeptide	-2,2346	2,151
MRE11A	MRE11 meiotic recombination 11 homolog A (S. cerevisiae)	-2,7895	1,9386
POLL	Polymerase (DNA directed), lambda	1,7901	1,5529
ACTB	Actin, beta	-1,3851	-1,3803
GAPDH	Glyceraldehyde-3-phosphate dehydrogenase	1,3851	1,3803

### Methotrexate-treatment leads to DNA repair signaling deregulation in SS cell lines

PCR array analysis after methotrexate-treatment in Hut-78 and SeAx cells revealed that methotrexate had a significant but lower, compared to bortezomib, impact on the DNA repair related genes’ expression (Figs 2 and 3), (Tables 1 and 2). Specifically, in Hut-78 cells (Fig 2A, 2B and 2D), the expression of one gene, *ERCC6*, was significantly up-regulated (more than 30-fold change), while 7 genes were more than 2-fold down-regulated compared to untreated cells (DSB: *XRCC6*, *XRCC4*, *PRKDC*, *RAD50*; NER: *RAD23A*, *CDK7* and SSBR related *ERCC1*). *ERCC1* gene’s expression presented the highest down-regulation, reaching a more than 600-fold change compared to untreated cells. Same wise, in SeAx cells the expression of several genes was affected after methotrexate treatment (Fig 3A, 3B and 3D). Six genes were up-regulated (*PRKDC*, *DDB2*, *XRCC3*, *XRCC1*, *RAD51B* and *ERCC6*) while two were down-regulated more than 2-fold (*CDK7* and *ATR*).

### Bortezomib-treatment effect on DNA repair signaling in MyLa cell line (MF)

**Fig 4:**
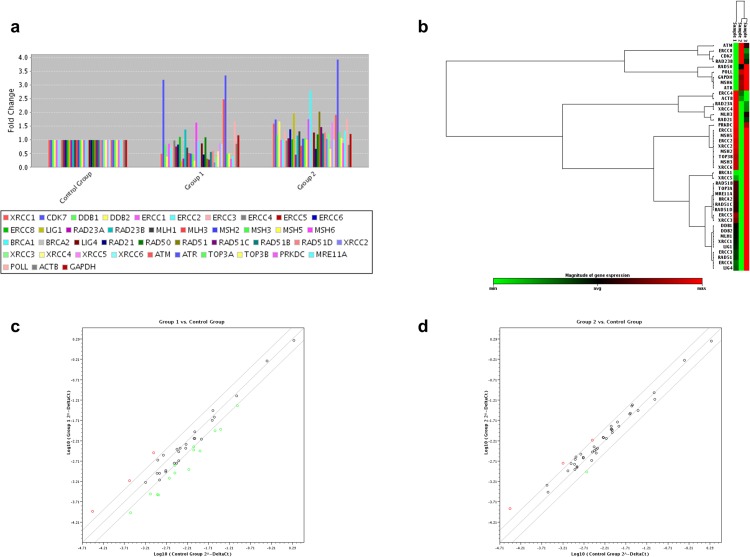
Differential gene expression in MyLa cell line determined by RT^2^ Profiler PCR arrays analysis after treatment with bortezomib and methotrexate. (a) Bar chart of the DNA repair related genes distribution after treatment with bortezomib (Group 1) and methotrexate (Group 2), (b) heat map of gene distribution after non-supervised hierarchical clustering of DNA repair related genes showing co-regulated genes across untreated (Sample 1), bortezomib-treated (Sample 2) and methotrexate-treated MyLa cells (Sample 3), (c) scatter plot of gene distribution after bortezomib-treatment (Group 1) and (d) after methotrexate-treatment (Group 2). In (a) each bar represents one gene. In (b) each box represents one gene, with minimum expression indicated as green, maximum as red and average as black. In the scatter plots (c) and (d) each circle represents one gene and up-regulated, down-regulated and unchanged genes are indicated as red, green and black, respectively.

Bortezomib-treatment of MyLa cell line followed by PCR arrays analysis revealed an altered expression profile of genes involved in the DNA repair pathways, compared to untreated cells (Fig 4A–4C), (Table 3). Specifically, bortezomib treatment led to a more than 2-fold change in the expression of a significant number of genes, with 3 genes being up-regulated (*ATR*, *CDK7* and *ATM*) and 14 genes down-regulated, involved in all DNA repair mechanisms tested (DSB: *RAD21*, *MRE11A*, *XRCC3*, *BRCA2*, *RAD51*, *PRKDC*, *RAD51C*, *XRCC2*; MMR: *MSH3*, *MSH5*; NER: *DDB2*, *RAD23A*, *LIG1*; SSBR: *XRCC1*) (Table 3), compared to untreated MyLa cells. *LIG1* and *XRCC2* genes showed the highest expression change, a more than 4-fold down-regulation.

**Table 3:** Fold changes of transcription in bortezomib- and methotrexate-treated MyLa cell line compared to that of untreated MyLa cell line.

Gene Symbol	Name of gene	Fold up- or down-regulation (Bortezomib-treated vs control)	Fold up- or down-regulation (Methotrexate-treated vs control)
XRCC1	X-ray repair complementing defective repair in Chinese hamster cells 1	-2,0069	1,5966
CDK7	Cyclin-dependent kinase 7	3,1932	1,7471
DDB1	Damage-specific DNA binding protein 1, 127kDa	-1,2184	1,1769
DDB2	Damage-specific DNA binding protein 2, 48kDa	-2,5403	1,676
ERCC1	Excision repair cross-complementing rodent repair deficiency, complementation group 1 (includes overlapping antisense sequence)	-1,1607	1,0035
ERCC2	Excision repair cross-complementing rodent repair deficiency, complementation group 2	-1,3803	1,0175
ERCC3	Excision repair cross-complementing rodent repair deficiency, complementation group 3 (xeroderma pigmentosum group B complementing)	-1,2702	1,3333
ERCC4	Excision repair cross-complementing rodent repair deficiency, complementation group 4	-1,0175	-1,0246
ERCC5	Excision repair cross-complementing rodent repair deficiency, complementation group 5	-1,3149	1,0607
ERCC6	Excision repair cross-complementing rodent repair deficiency, complementation group 6	-1,2016	1,3899
ERCC8	Excision repair cross-complementing rodent repair deficiency, complementation group 8	1,1134	1,0246
LIG1	Ligase I, DNA, ATP-dependent	-4,4229	1,9656
RAD23A	RAD23 homolog A (S. cerevisiae)	-3,1275	-2,166
RAD23B	RAD23 homolog B (S. cerevisiae)	1,3803	1,1607
MLH1	MutL homolog 1, colon cancer, nonpolyposis type 2 (E. coli)	-1,3899	1,3149
MLH3	MutL homolog 3 (E. coli)	-1,9252	-1,2702
MSH2	MutS homolog 2, colon cancer, nonpolyposis type 1 (E. coli)	-1,9793	1,0461
MSH3	MutS homolog 3 (E. coli)	-2,0209	1,0607
MSH5	MutS homolog 5 (E. coli)	-4,0982	1,0105
MSH6	MutS homolog 6 (E. coli)	1,6301	1,7593
BRCA1	Breast cancer 1, early onset	-1,0607	2,7992
BRCA2	Breast cancer 2, early onset	-2,6851	1,3803
LIG4	Ligase IV, DNA, ATP-dependent	-1,1447	1,2702
RAD21	RAD21 homolog (S. pombe)	-2,1361	-1,4794
RAD50	RAD50 homolog (S. cerevisiae)	1,0981	1,2016
RAD51	RAD51 homolog (S. cerevisiae)	-3,1492	2,035
RAD51C	RAD51 homolog C (S. cerevisiae)	-3,5677	1,4692
RAD51B	RAD51 homolog B (S. cerevisiae)	-1,7839	1,2354
RAD51D	RAD51 homolog D (S. cerevisiae)	-1,676	1,2702
XRCC2	X-ray repair complementing defective repair in Chinese hamster cells 2	-5,5983	1,0389
XRCC3	X-ray repair complementing defective repair in Chinese hamster cells 3	-2,5937	1,2269
XRCC4	X-ray repair complementing defective repair in Chinese hamster cells 4	-1,7112	-1,4794
XRCC5	X-ray repair complementing defective repair in Chinese hamster cells 5 (double-strand-break rejoining)	-1,1447	1,6415
XRCC6	X-ray repair complementing defective repair in Chinese hamster cells 6	-1,1607	1,0175
ATM	Ataxia telangiectasia mutated	2,488	1,9119
ATR	Ataxia telangiectasia and Rad3 related	3,3519	3,9313
TOP3A	Topoisomerase (DNA) III alpha	-1,9793	1,279
TOP3B	Topoisomerase (DNA) III beta	-1,9793	1,0755
PRKDC	Protein kinase, DNA-activated, catalytic polypeptide	-3,2378	-1,1212
MRE11A	MRE11 meiotic recombination 11 homolog A (S. cerevisiae)	-2,4033	1,3241
POLL	Polymerase (DNA directed), lambda	1,6876	1,7593
ACTB	Actin, beta	-1,1688	-1,2184
GAPDH	Glyceraldehyde-3-phosphate dehydrogenase	1,1688	1,2184

### Methotrexate-treatment effect on DNA repair signaling in MyLa cell line (MF)

Methotrexate-treatment of MyLa cell line had a rather moderate effect on the expression profile of the selected DNA repair related genes, compared to untreated cells, as revealed through PCR arrays analysis (Fig 4A, 4B and 4D). We found that only a total of 4 genes were significantly affected (Table 3). Specifically, *ATR*, *BRCA1* and *RAD51* genes were up-regulated more than 2-fold, while the expression of *RAD23A* (NER mechanism) was down-regulated.

## Discussion

Although many chemotherapeutic agents have been used in the treatment of MF/SS, no single therapy has shown superior or efficient effect in the long term, especially for refractory, extensive and/or advanced disease, which are mostly approached through systemic treatments that also fail to provide a permanent therapeutic solution [33]. Since there are no current curative treatment options for CTCL patients, there is an even higher necessity for a better insight into the pathogenesis of the disease.

We investigated for the first time whether two well-known therapeutic agents, bortezomib and methotrexate, interfere with the DNA repair mechanisms in CTCL cell lines and manage to overcome their resistance to apoptosis.

We initially tested the ability of the aforementioned agents and their combination to induce apoptosis in SS (Hut-78 and SeAx) and MF (MyLa) cell lines. Our data demonstrate that both SS cell lines (Hut-78 and SeAx) respond with statistically enhanced apoptosis after all three treatments, with bortezomib presenting the highest effect. The MF cell line MyLa responded with a statistically significant but rather moderate augmentation in apoptosis only after treatment with each agent separately and not their combination, with methotrexate presenting the higher apoptotic impact between the two. Our results are in accordance with previously published studies, since both agents have been reported to induce apoptosis in CTCL through various mechanisms. Specifically, methotrexate has been shown to induce apoptosis in MF/SS cell lines through the enhancement of FAS expression in cell lines and leukemic cells by inhibiting methylation of the FAS promoter and thereby de-repressing gene expression, resulting in the upregulation of proteins essential to T-cell apoptosis [34, 35]. Bortezomib has also been reported to induce apoptosis in CTCL cell lines. Previous studies have shown that bortezomib induces nuclear translocation of IkBa resulting in gene-specific suppression of NF-kB–dependent transcription and induction of apoptosis in CTCL [36, 37]. Moreover, bortezomib induces CTCL cell apoptosis by inhibiting the expression of the antiapoptotic genes *cIAP1* and *cIAP2* and the proto-oncogene *Bcl3* [38] while it also suppresses the TGF-b1 cytokine leading to apoptosis and IL-8 and IL-17 expression and inhibits the *CXCR4* chemokine receptor’s expression, resulting in a decreased TGF-β1-mediated CTCL cells migration [39]. Apart from verifying the apoptotic effect of bortezomib and methotrexate on CTCL, our results reveal for the first time the effectiveness of each agent and/or their combination in SS and MF cell lines. Specifically, our observations indicate that bortezomib is the most efficient agent in SS cell lines in terms of apoptosis and methotrexate in MF cells, while the two drugs do not seem to act synergistically *in vitro*, suggesting lack of efficacy of this combination in clinical use.

We next investigated the effect of bortezomib- and methotrexate- treatment on DNA repair signaling pathways in CTCL using PCR arrays technology. Our data demonstrate for the first time that both drugs significantly alter the expression of a large number of genes involved in DNA repair systems in SS and MF cell lines, suggesting an additional possible mechanism for the enhanced apoptosis of CTCL after treatment with the aforementioned agents. These observations are further supported by an increasing number of studies which indicate a connection between aberrant DNA repair mechanisms and the pathogenesis of CTCL. Specifically, a recent study based on whole exome and SNP array based copy number analyses of CD4^+^ tumour cells of a large number of untreated SS patients showed various aberrations in genes related to DNA repair and genome stability, such as the *POT1* and *ATM* genes responsible for telomere maintenance and genes associated to homologous recombination (*RAD51C*, *BRCA2*, *POLD1*) [40]. In addition, the aforementioned association between aberrant DNA repair mechanisms and CTCL pathogenesis has been demonstrated through the identification of a novel CTCL cell line, BKP1, derived from the peripheral blood of a SS patient. BKP1 cells bear a deletion of three genes involved in the repair of DNA damage (*FANCC*, *FANCM* and *XPA*), suggesting that this could lead to genomic instability [41]. Moreover, there is evidence that promoter hypermethylation in CTCL leads, among others, to deregulation of *MGMT*, a gene that encodes a DNA repair enzyme responsible for the removal of alkylating lesions at the guanine base protecting against mutagenesis and malignant transformation [42]. Therefore, DNA repair related deficiencies could be associated with the reported apoptotic resistance of CTCL and the related pathways should be considered as possible therapeutic targets.

Most significantly, our data reveal that each agent has a different effect in terms of apoptosis’ induction and DNA repair profile alterations on SS and MF cell lines. Specifically, in both SS cell lines, bortezomib induced higher apoptotic rates and presented with a greater impact on DNA repair profile than methotrexate. Interestingly, in the MF cell line MyLa, methotrexate and not bortezomib appeared with the higher impact on apoptosis while the opposite was observed in terms of DNA repair genes’ expression, where bortezomib seemed to affect more genes than methotrexate. These findings could be attributed to the differences in the mechanism of action between bortezomib and methotrexate and are in accordance with previously published reports. Indeed, bortezomib has been previously associated with the regulation of DNA damage response (DDR). Specifically, the mechanisms of proteasome inhibition’s lethality have been reviewed in the past and include, among others, the inhibition of the DNA repair mechanisms [43]. Moreover, the role of the proteasome in the DNA repair response has already been proposed [44, 45], while proteasome inhibitors have been reported to sensitize tumour cells to DNA-damaging agents and cause a deficient DNA repair [46, 47]. Howard and colleagues [48], examined the effect of bortezomib on Double strand break (DSB) repair and suggested that the influence of bortezomib on the DDR and hence on DNA crosslink sensitivity, disrupts FANC signalling and possibly blocks Alternative end joining (Alt-EJ), homologous recombination (HR), and/or end resection. There are fewer reports regarding the association of methotrexate with DNA repair systems. Nonetheless, methotrexate has been shown to affect the homologous recombination repair of DNA DSBs by controlling the expression of HR related genes and suppressing the proper assembly of HR recombination-directed subnuclear foci [49].

Our data further demonstrate that the examined SS cell lines presented similarities in their response to each agent in terms of DNA repair pathways’ alterations, while the MF cell line exhibited a different profile. These observations are in line with the distinct clinical nature of SS and MF, since these diseases arise from functionally and phenotypically different cell populations, as previously reported [8, 50]. Specifically, the malignant T cells in SS/L-CTCL express markers of central memory T cells (T_CM_) and therefore SS is a malignancy of T_CM_, while those in MF express markers of skin resident effector memory T cells (T_EM_), suggesting that MF is a malignancy of T_EM_ cells [8, 51]. In addition, Hut-78, SeAx and MyLa cell lines bear differences regarding their p53 status, as revealed after p53 mutation analysis. Hut-78 cells bear a homozygous nonsense mutation, SeAx a homozygous missense mutation and MyLa (MF cell line) was shown to carry wild-type p53 [52]. Therefore, in contrast to MyLa cell line, both SS cell lines lack a functional p53, which could be associated with the observed different response profile of SS and MF cell lines, given the importance of p53 in multiple cellular pathways. Indeed, p53 is a tumour-suppressor protein which responds to a wide variety of stress signals including DNA damage and ultimately decides on the cellular fate through activation of cell cycle progression, apoptosis, senescence, metabolism, and autophagy [53, 54], depending on the nature of the stress signals, the type of tissue and the cellular environment [55]. The above suggest that the increased apoptotic rate of the SS cell lines after treatment with both drugs could be initiated by p53-independent pathways which compensate for p53 function, while for the MF cell line the observed apoptosis could be p53 induced, thus explaining the differential response between SS and MF. This notion is further supported by different studies showing that bortezomib is capable of inducing apoptotic cell death in both a p53-dependent and a p53-independent manner [56–58]. Moreover, methotrexate has been shown to induce apoptosis through a p53/p21- dependent pathway [59, 60]. This could further explain the reason why the wild-type p53 MyLa cell line exhibited higher apoptotic rates under methotrexate-treatment compared to bortezomib-treatment. In contrast, the p53 deficient SS cell lines respond with the higher level of apoptosis after bortezomib treatment and not methotrexate, since the proteasome inhibitor can probably initiate a p53-independent cell death program and therefore effectively induce apoptosis. Based on the above, CTCL patients, both SS and MF, could possibly benefit from a combinational treatment in a clinical level. The apoptotic rate of bortezomib in SS could be augmented by a co-treatment with agents that act in a p53 dependent manner in terms of apoptosis induction, whereas methotrexate treatment in MF could lead to an enhanced apoptotic rate when combined with an agent that acts through a p53 independent manner, apoptosis-wise. However, according to our data, the combination of bortezomib and methotrexate does not manage to achieve higher death rates than monotherapy and therefore is not suggested for clinical use in either SS or MF. Further studies, initially in an *in vitro* level, are required in order to reveal the most successful combinations for overcoming the CTCL’s resistance to chemotherapy.

In summary, we revealed that bortezomib and methotrexate induce DNA repair systems’ deregulation in MF and SS suggesting another mechanism through which these agents lead to apoptosis. We also provided clinically significant information regarding CTCL therapy, showing *in vitro* that bortezomib is a more suitable option for the treatment of SS while methotrexate for MF. Finally, we showed that the bortezomib/methotrexate combination fails to achieve better responses compared to monotherapy in CTCL cell lines, indicating that there is no rationale for such a chemotherapeutic combination in our clinical practice.

Our observations need to be further confirmed following the results of clinical trials in CTCL patients, where the distinct nature of each condition, SS or MF, should be taken into consideration.

## References

[1] Girardi M, Heald PW, Wilson LD. The pathogenesis of mycosis fungoides. N Engl J Med. 2004;350(19):1978–88. 10.1056/NEJMra032810 15128898

[2] Devata S, Wilcox RA. Cutaneous T-Cell Lymphoma: A Review with a Focus on Targeted Agents. Am J Clin Dermatol. 2016. 10.1007/s40257-016-0177-5 26923912

[3] Willemze R, Jaffe ES, Burg G, Cerroni L, Berti E, Swerdlow SH, et al WHO-EORTC classification for cutaneous lymphomas. Blood. 2005;105(10):3768–85. 10.1182/blood-2004-09-3502 15692063

[4] Kim YH, Liu HL, Mraz-Gernhard S, Varghese A, Hoppe RT. Long-term outcome of 525 patients with mycosis fungoides and Sezary syndrome: clinical prognostic factors and risk for disease progression. Arch Dermatol. 2003;139(7):857–66. 10.1001/archderm.139.7.857 12873880

[5] Li JY, Horwitz S, Moskowitz A, Myskowski PL, Pulitzer M, Querfeld C. Management of cutaneous T cell lymphoma: new and emerging targets and treatment options. Cancer Manag Res. 2012;4:75–89. PubMed Central PMCID: PMCPMC3308634. 10.2147/CMAR.S9660 22457602 PMC3308634

[6] Drews RE. Emerging treatment options for advanced-stage mycosis fungoides. J Clin Oncol. 2012;30(33):4064–70. 10.1200/JCO.2012.44.5650 23045584

[7] Navi D, Riaz N, Levin YS, Sullivan NC, Kim YH, Hoppe RT. The Stanford University experience with conventional-dose, total skin electron-beam therapy in the treatment of generalized patch or plaque (T2) and tumor (T3) mycosis fungoides. Arch Dermatol. 2011;147(5):561–7. 10.1001/archdermatol.2011.98 21576575

[8] Chung CG, Poligone B. Cutaneous T cell Lymphoma: an Update on Pathogenesis and Systemic Therapy. Curr Hematol Malig Rep. 2015;10(4):468–76. 10.1007/s11899-015-0293-y 26626770

[9] Hideshima T, Richardson P, Chauhan D, Palombella VJ, Elliott PJ, Adams J, et al The proteasome inhibitor PS-341 inhibits growth, induces apoptosis, and overcomes drug resistance in human multiple myeloma cells. Cancer Res. 2001;61(7):3071–6. Epub 2001/04/18. 11306489

[10] Shah JJ, Orlowski RZ. Proteasome inhibitors in the treatment of multiple myeloma. Leukemia. 2009;23(11):1964–79. PubMed Central PMCID: PMCPMC4737506. 10.1038/leu.2009.173 19741722 PMC4737506

[11] Jain S, Zain J, O'Connor O. Novel therapeutic agents for cutaneous T-Cell lymphoma. J Hematol Oncol. 2012;5:24 PubMed Central PMCID: PMCPMC3418166. 10.1186/1756-8722-5-24 22594538 PMC3418166

[12] Buac D, Shen M, Schmitt S, Kona FR, Deshmukh R, Zhang Z, et al From bortezomib to other inhibitors of the proteasome and beyond. Curr Pharm Des. 2013;19(22):4025–38. PubMed Central PMCID: PMCPMC3657018. 23181572 10.2174/1381612811319220012 PMC3657018

[13] Orlowski RZ, Kuhn DJ. Proteasome inhibitors in cancer therapy: lessons from the first decade. Clin Cancer Res. 2008;14(6):1649–57. 10.1158/1078-0432.CCR-07-2218 18347166

[14] Kozuch PS, Rocha-Lima CM, Dragovich T, Hochster H, O'Neil BH, Atiq OT, et al Bortezomib with or without irinotecan in relapsed or refractory colorectal cancer: results from a randomized phase II study. J Clin Oncol. 2008;26(14):2320–6. 10.1200/JCO.2007.14.0152 18467723

[15] Morris MJ, Kelly WK, Slovin S, Ryan C, Eicher C, Heller G, et al A phase II trial of bortezomib and prednisone for castration resistant metastatic prostate cancer. J Urol. 2007;178(6):2378–83; discussion 83–4. 10.1016/j.juro.2007.08.015 17936848

[16] Schmid P, Kuhnhardt D, Kiewe P, Lehenbauer-Dehm S, Schippinger W, Greil R, et al A phase I/II study of bortezomib and capecitabine in patients with metastatic breast cancer previously treated with taxanes and/or anthracyclines. Ann Oncol. 2008;19(5):871–6. 10.1093/annonc/mdm569 18209010

[17] Heider U, Rademacher J, Lamottke B, Mieth M, Moebs M, von Metzler I, et al Synergistic interaction of the histone deacetylase inhibitor SAHA with the proteasome inhibitor bortezomib in cutaneous T cell lymphoma. Eur J Haematol. 2009;82(6):440–9. 10.1111/j.1600-0609.2009.01239.x 19220424

[18] Horwitz SM. Novel therapies for cutaneous T-cell lymphomas. Clin Lymphoma Myeloma. 2008;8 Suppl 5:S187–92. 19073526 10.3816/CLM.2008.s.015

[19] Kim SJ, Yoon DH, Kang HJ, Kim JS, Park SK, Kim HJ, et al Bortezomib in combination with CHOP as first-line treatment for patients with stage III/IV peripheral T-cell lymphomas: a multicentre, single-arm, phase 2 trial. Eur J Cancer. 2012;48(17):3223–31. 10.1016/j.ejca.2012.06.003 22770877

[20] Zinzani PL, Musuraca G, Tani M, Stefoni V, Marchi E, Fina M, et al Phase II trial of proteasome inhibitor bortezomib in patients with relapsed or refractory cutaneous T-cell lymphoma. J Clin Oncol. 2007;25(27):4293–7. 10.1200/JCO.2007.11.4207 17709797

[21] Biskup E, Kamstrup MR, Manfe V, Gniadecki R. Proteasome inhibition as a novel mechanism of the proapoptotic activity of gamma-secretase inhibitor I in cutaneous T-cell lymphoma. Br J Dermatol. 2013;168(3):504–12. 10.1111/bjd.12071 23445313

[22] Morales C, Garcia MJ, Ribas M, Miro R, Munoz M, Caldas C, et al Dihydrofolate reductase amplification and sensitization to methotrexate of methotrexate-resistant colon cancer cells. Mol Cancer Ther. 2009;8(2):424–32. 10.1158/1535-7163.MCT-08-0759 19190117

[23] Lorico A, Toffoli G, Boiocchi M, Erba E, Broggini M, Rappa G, et al Accumulation of DNA strand breaks in cells exposed to methotrexate or N10-propargyl-5,8-dideazafolic acid. Cancer Res. 1988;48(8):2036–41. 3349474

[24] Barnhart K, Coutifaris C, Esposito M. The pharmacology of methotrexate. Expert Opin Pharmacother. 2001;2(3):409–17. 10.1517/14656566.2.3.409 11336595

[25] Olsen EA. The pharmacology of methotrexate. J Am Acad Dermatol. 1991;25(2 Pt 1):306–18. 1918470 10.1016/0190-9622(91)70199-c

[26] Aviles A, Nambo MJ, Neri N, Castaneda C, Cleto S, Gonzalez M, et al Interferon and low dose methotrexate improve outcome in refractory mycosis fungoides/Sezary syndrome. Cancer Biother Radiopharm. 2007;22(6):836–40. 10.1089/cbr.2007.0402 18158775

[27] Aviles A, Neri N, Fernandez-Diez J, Silva L, Nambo MJ. Interferon and low doses of methotrexate versus interferon and retinoids in the treatment of refractory/relapsed cutaneous T-cell lymphoma. Hematology. 2015;20(9):538–42. 10.1179/1607845415Y.0000000002 25592781

[28] Kannangara AP, Levitan D, Fleischer AB Jr. Evaluation of the efficacy of the combination of oral bexarotene and methotrexate for the treatment of early stage treatment-refractory cutaneous T-cell lymphoma. J Dermatolog Treat. 2009;20(3):169–76. 10.1080/09546630802562427 19016373

[29] Wright JC, Lyons MM, Walker DG, Golomb FM, Gumport SL, Medrek TJ. Observations on the Use of Cancer Chemotherapeutic Agents in Patients with Mycosis Fungoides. Cancer. 1964;17:1045–62. 14202592 10.1002/1097-0142(196408)17:8<1045::aid-cncr2820170811>3.0.co;2-s

[30] Zackheim HS, Kashani-Sabet M, Hwang ST. Low-dose methotrexate to treat erythrodermic cutaneous T-cell lymphoma: results in twenty-nine patients. J Am Acad Dermatol. 1996;34(4):626–31. 8601652 10.1016/s0190-9622(96)80062-4

[31] Zackheim HS, Kashani-Sabet M, McMillan A. Low-dose methotrexate to treat mycosis fungoides: a retrospective study in 69 patients. J Am Acad Dermatol. 2003;49(5):873–8. 14576667 10.1016/s0190-9622(03)01591-3

[32] Mpakou VE, Kontsioti F, Papageorgiou S, Spathis A, Kottaridi C, Girkas K, et al Dasatinib inhibits proliferation and induces apoptosis in the KASUMI-1 cell line bearing the t(8;21)(q22;q22) and the N822K c-kit mutation. Leukemia research. 2013;37(2):175–82. 10.1016/j.leukres.2012.10.011 23149070

[33] Hughes CF, Khot A, McCormack C, Lade S, Westerman DA, Twigger R, et al Lack of durable disease control with chemotherapy for mycosis fungoides and Sezary syndrome: a comparative study of systemic therapy. Blood. 2015;125(1):71–81. 10.1182/blood-2014-07-588236 25336628

[34] Wu J, Nihal M, Siddiqui J, Vonderheid EC, Wood GS. Low FAS/CD95 expression by CTCL correlates with reduced sensitivity to apoptosis that can be restored by FAS upregulation. J Invest Dermatol. 2009;129(5):1165–73. 10.1038/jid.2008.309 18923451

[35] Wu J, Wood GS. Reduction of Fas/CD95 promoter methylation, upregulation of Fas protein, and enhancement of sensitivity to apoptosis in cutaneous T-cell lymphoma. Arch Dermatol. 2011;147(4):443–9. 10.1001/archdermatol.2010.376 21173302

[36] Juvekar A, Manna S, Ramaswami S, Chang TP, Vu HY, Ghosh CC, et al Bortezomib induces nuclear translocation of IkappaBalpha resulting in gene-specific suppression of NF-kappaB—dependent transcription and induction of apoptosis in CTCL. Mol Cancer Res. 2011;9(2):183–94. PubMed Central PMCID: PMCPMC3078042. 10.1158/1541-7786.MCR-10-0368 21224428 PMC3078042

[37] Sors A, Jean-Louis F, Pellet C, Laroche L, Dubertret L, Courtois G, et al Down-regulating constitutive activation of the NF-kappaB canonical pathway overcomes the resistance of cutaneous T-cell lymphoma to apoptosis. Blood. 2006;107(6):2354–63. 10.1182/blood-2005-06-2536 16219794

[38] Chang TP, Vancurova I. Bcl3 regulates pro-survival and pro-inflammatory gene expression in cutaneous T-cell lymphoma. Biochim Biophys Acta. 2014;1843(11):2620–30. PubMed Central PMCID: PMCPMC4158825. 10.1016/j.bbamcr.2014.07.012 25089799 PMC4158825

[39] Chang TP, Poltoratsky V, Vancurova I. Bortezomib inhibits expression of TGF-beta1, IL-10, and CXCR4, resulting in decreased survival and migration of cutaneous T cell lymphoma cells. J Immunol. 2015;194(6):2942–53. PubMed Central PMCID: PMCPMC4355060. 10.4049/jimmunol.1402610 25681335 PMC4355060

[40] Woollard WJ, Pullabhatla V, Lorenc A, Patel VM, Butler RM, Bayega A, et al Candidate driver genes in Sezary syndrome: frequent perturbations of genes involved in genome maintenance and DNA repair. Blood. 2016. 10.1182/blood-2016-02-699843 27121473

[41] Boudjarane J, Essaydi A, Farnault L, Popovici C, Lafage-Pochitaloff M, Beaufils N, et al Characterization of the novel Sezary lymphoma cell line BKP1. Exp Dermatol. 2015;24(1):60–2. 10.1111/exd.12567 25314094

[42] van Doorn R, Zoutman WH, Dijkman R, de Menezes RX, Commandeur S, Mulder AA, et al Epigenetic profiling of cutaneous T-cell lymphoma: promoter hypermethylation of multiple tumor suppressor genes including BCL7a, PTPRG, and p73. J Clin Oncol. 2005;23(17):3886–96. 10.1200/JCO.2005.11.353 15897551

[43] Holkova B, Grant S. Proteasome inhibitors in mantle cell lymphoma. Best Pract Res Clin Haematol. 2012;25(2):133–41. PubMed Central PMCID: PMCPMC3374152. 10.1016/j.beha.2012.04.007 22687449 PMC3374152

[44] Krogan NJ, Lam MH, Fillingham J, Keogh MC, Gebbia M, Li J, et al Proteasome involvement in the repair of DNA double-strand breaks. Mol Cell. 2004;16(6):1027–34. 10.1016/j.molcel.2004.11.033 15610744

[45] Motegi A, Murakawa Y, Takeda S. The vital link between the ubiquitin-proteasome pathway and DNA repair: impact on cancer therapy. Cancer Lett. 2009;283(1):1–9. 10.1016/j.canlet.2008.12.030 19201084

[46] Jacquemont C, Taniguchi T. Proteasome function is required for DNA damage response and fanconi anemia pathway activation. Cancer Res. 2007;67(15):7395–405. 10.1158/0008-5472.CAN-07-1015 17671210

[47] Takeshita T, Wu W, Koike A, Fukuda M, Ohta T. Perturbation of DNA repair pathways by proteasome inhibitors corresponds to enhanced chemosensitivity of cells to DNA damage-inducing agents. Cancer Chemother Pharmacol. 2009;64(5):1039–46. PubMed Central PMCID: PMCPMC2728221. 10.1007/s00280-009-0961-5 19274461 PMC2728221

[48] Howard SM, Yanez DA, Stark JM. DNA damage response factors from diverse pathways, including DNA crosslink repair, mediate alternative end joining. PLoS Genet. 2015;11(1):e1004943 PubMed Central PMCID: PMCPMC4309583. 10.1371/journal.pgen.1004943 25629353 PMC4309583

[49] Du LQ, Du XQ, Bai JQ, Wang Y, Yang QS, Wang XC, et al Methotrexate-mediated inhibition of RAD51 expression and homologous recombination in cancer cells. J Cancer Res Clin Oncol. 2012;138(5):811–8. 10.1007/s00432-011-1132-8 22274865 PMC11824462

[50] Sallusto F, Lenig D, Forster R, Lipp M, Lanzavecchia A. Two subsets of memory T lymphocytes with distinct homing potentials and effector functions. Nature. 1999;401(6754):708–12. 10.1038/44385 10537110

[51] Campbell JJ, Clark RA, Watanabe R, Kupper TS. Sezary syndrome and mycosis fungoides arise from distinct T-cell subsets: a biologic rationale for their distinct clinical behaviors. Blood. 2010;116(5):767–71. PubMed Central PMCID: PMCPMC2918332. 10.1182/blood-2009-11-251926 20484084 PMC2918332

[52] Manfe V, Biskup E, Johansen P, Kamstrup MR, Krejsgaard TF, Morling N, et al MDM2 inhibitor nutlin-3a induces apoptosis and senescence in cutaneous T-cell lymphoma: role of p53. J Invest Dermatol. 2012;132(5):1487–96. 10.1038/jid.2012.10 22377766

[53] Kruiswijk F, Labuschagne CF, Vousden KH. p53 in survival, death and metabolic health: a lifeguard with a licence to kill. Nat Rev Mol Cell Biol. 2015;16(7):393–405. 10.1038/nrm4007 26122615

[54] Vousden KH, Lane DP. p53 in health and disease. Nat Rev Mol Cell Biol. 2007;8(4):275–83. 10.1038/nrm2147 17380161

[55] Vousden KH, Prives C. Blinded by the Light: The Growing Complexity of p53. Cell. 2009;137(3):413–31. 10.1016/j.cell.2009.04.037 19410540

[56] Lopes UG, Erhardt P, Yao R, Cooper GM. p53-dependent induction of apoptosis by proteasome inhibitors. J Biol Chem. 1997;272(20):12893–6. Epub 1997/05/16. 9148891 10.1074/jbc.272.20.12893

[57] Yerlikaya A, Okur E, Baykal AT, Acilan C, Boyaci I, Ulukaya E. A proteomic analysis of p53-independent induction of apoptosis by bortezomib in 4T1 breast cancer cell line. J Proteomics. 2015;113:315–25. 10.1016/j.jprot.2014.09.010 25305590

[58] Yerlikaya A, Okur E, Ulukaya E. The p53-independent induction of apoptosis in breast cancer cells in response to proteasome inhibitor bortezomib. Tumour Biol. 2012;33(5):1385–92. 10.1007/s13277-012-0386-3 22477712

[59] Gibson RJ, Bowen JM, Cummins AG, Keefe DM. Relationship between dose of methotrexate, apoptosis, p53/p21 expression and intestinal crypt proliferation in the rat. Clin Exp Med. 2005;4(4):188–95. 10.1007/s10238-004-0055-y 15750766

[60] Huang WY, Yang PM, Chang YF, Marquez VE, Chen CC. Methotrexate induces apoptosis through p53/p21-dependent pathway and increases E-cadherin expression through downregulation of HDAC/EZH2. Biochem Pharmacol. 2011;81(4):510–7. 10.1016/j.bcp.2010.11.014 21114963

